# Elastic–plastic model identification for rock surrounding an underground excavation based on immunized genetic algorithm

**DOI:** 10.1186/s40064-016-2726-z

**Published:** 2016-07-11

**Authors:** Wei Gao, Dongliang Chen, Xu Wang

**Affiliations:** Key Laboratory of Ministry of Education for Geomechanics and Embankment Engineering, College of Civil and Transportation Engineering, Hohai University, 1 Xikang Road, Nanjing, 210098 China

**Keywords:** Elastic–plastic constitutive model, Surrounding rock, Identification, Immunized genetic algorithm, Underground engineering

## Abstract

To compute the stability of underground engineering, a constitutive model of surrounding rock must be identified. Many constitutive models for rock mass have been proposed. In this model identification study, a generalized constitutive law for an elastic–plastic constitutive model is applied. Using the generalized constitutive law, the problem of model identification is transformed to a problem of parameter identification, which is a typical and complicated optimization. To improve the efficiency of the traditional optimization method, an immunized genetic algorithm that is proposed by the author is applied in this study. In this new algorithm, the principle of artificial immune algorithm is combined with the genetic algorithm. Therefore, the entire computation efficiency of model identification will be improved. Using this new model identification method, a numerical example and an engineering example are used to verify the computing ability of the algorithm. The results show that this new model identification algorithm can significantly improve the computation efficiency and the computation effect.

## Background

The back analysis method is an important method in underground engineering. Since it was proposed in the 1970s, numerous studies have been performed (Gao and Liu 2009; Wang and Li 1993; Sakurai and Takeuchi 1983). Currently, back analysis can be divided into two types of analyses: parameter identification and model identification (Gao and Liu 2009). In parameter identification, the constitutive model of surrounding rock is assumed to be a simple model, such as the elastic model, the elastic–plastic model and the rheological model. Using these simple models, the mechanical parameters of surrounding rock can be inversed based on the measurement information, such as displacement. For simplicity, parameter identification based on measured displacements has been the most common back analysis method for underground engineering (Feng et al. 2000; Maier and Gioda 1982; Rechea et al. 2008; Sharifzadeh et al. 2013; Yazdani et al. 2012). However, due to its complexity, model identification has developed very slowly. Different from parameter identification, in model identification, the constitutive model of surrounding rock is unknown. Based on the measurement information, such as displacement, the structure and parameters of the constitutive model can be identified. Because it is very complex to select the structure and parameters of the constitutive model at the same time, generally, the structure of the constitutive model is determined by the prior knowledge, such as engineering experience, and then only the parameters of the constitutive model are identified based on measurement information. Thus, the model identification can be simplified as similar as the parameter identification. However, the model identification is more complex than the parameter identification. In model identification, the model parameters and the mechanical parameters can be identified at the same time. And the structure of the constitutive model determined by the prior knowledge is generally more complex than the assumed simple model used in parameter identification. Moreover, different from the mechanical parameters, which have the clear physical and mechanical meaning, and can be determined by the tests easily, the model parameters, which only describe the constitutive model, can not be determined by the tests. In 1987, Gioda and Sakurai proposed that model identification based on displacement should be the main development for back analysis (Gioda and Sakurai 1987). In 1997, Sakurai demonstrated that the identification of a constitutive model is critical (Sakurai 1997). Therefore, researchers have investigated the identification of a constitutive model. Based on the displacement measurements of an underground roadway, Liu (2011) identified the visco-elastic constitutive model of rock mass based on the traditional nonlinear optimum technique. Wang et al. (2007) identified the geo-material constitutive model based on their constitutive model database and several identification algorithms. Yang and Wang (2009) presented a numerical model to identify the unknown equivalent constitutive model in the elastic layered rock mass of an underground opening by the Gauss–Newton technique.

**Fig. 1:**
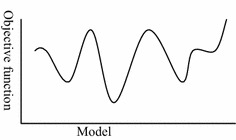
Optimization objective function of model identification for underground engineering

**Fig. 2:**
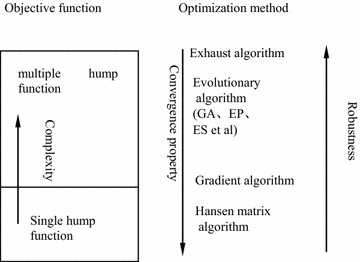
Relationship between objective function and optimization method

Because model identification is a very complicated optimization problem (Gao and Liu 2009), whose objective function can be shown as in Fig. 1, traditional optimization techniques have some shortcomings. The relationship between objective function and the optimization method can be described as in Fig. 2 (Gao and Liu 2009). From Fig. 2, the global optimization is one suitable method to solve model identification problem. Therefore, some important contributions to global optimization algorithms have been achieved. Su et al. (2008) identified the structure and parameters of the rheological constitutive model of the surrounding rocks of the Jinping tunnels in China using a differential evolution algorithm. Feng et al. (2006) identified a visco-elastic model of surrounding rocks in the Goupitan hydroelectric power station in China using genetic programming and a modified particle swarm optimization algorithm. Meier et al. (2007) have performed model identification of surrounding rocks for tunneling by particle swarm optimization. Sha et al. (2011) presented three methods to identify the constitutive model of rocks based on a genetic algorithm, a back propagation neural network and genetic programming and compared all methods. Surajit and Wathugala (1996) calibrated a constitutive model using genetic algorithms. Gao (2007) proposed a method to identify the rheological constitutive model for rock mass of an underground roadway based on the fast-convergent genetic algorithm. Moreover, Gao et al. (2004) presented an identification method for the surrounding rock of an underground power house based on a new intelligent bionics algorithm. Because these global optimization algorithms comprise random search algorithms, their computational efficiency is very low. This low efficiency is problematic when the model identification is extremely complicated. In this study, a new method to identify the constitutive model based on an immunized genetic algorithm is proposed. The elastic–plastic constitutive model is the main model of geo-materials which has been comprehensively evaluated (Nakai 2012; Zheng et al. 2002). In the last years, many studies (Cui et al. 2015; Lee and Pietruszczak 2008; Spiezia et al. 2016; Zhang et al. 2012) have been carried out investigating the elasto-plastic behavior of rock surrounding underground excavations. And those researches have proved that the elastic–plastic constitutive model can describe the mechanical behaviour of rock surrounding underground excavation very well. Therefore, the elastic–plastic constitutive model is analyzed in this study.

## Generalized constitutive law of elastic–plastic model for rock materials

The generalized constitutive law of surrounding rock in strain space is

1 $$\left\{ {\begin{array}{ll} {d\sigma_{ij} = D_{ijkl} d\varepsilon_{kl} - \frac{1}{A}D_{ijmn} \frac{\partial g}{{\partial \sigma_{mn} }}\left\langle {\frac{\partial F}{{\partial \varepsilon_{kl} }}d\varepsilon_{ij} } \right\rangle } \hfill \\ {d\sigma_{ij} = D_{ijkl} d\varepsilon_{kl} } \hfill \\ \end{array} } \right. \quad {\text{if}}\quad \left\{ {\begin{array}{ll} f = 0 \\ f < 0 \\ \end{array} } \right.$$

where *D*_*ijkl*_ is the tensor of the elastic stiffness coefficient, *g*(*σ*_*ij*_, *k*) is the plastic potential function, *f*(*σ*_*ij*_, *σ*_*ij*_^*p*^, *k*) is the yield function of the stress space, *F*(*ɛ*_*ij*_, *ɛ*_*ij*_^*p*^, *k*) is the yield function of the strain space, *σ*_*ij*_^*p*^ and *ɛ*_*ij*_^*p*^ the plastic stress and strain, respectively, *A* is the hardening or softening parameter, whose form is as follows (Zheng et al. 2002; Zheng and Kong 2010):

2 $$A = \frac{\partial F}{{\partial \varepsilon_{ij} }} \cdot \frac{\partial g}{{\partial \sigma_{kl} }} - \frac{\partial f}{\partial k}h$$

*k* is a plastic internal variable, *h* is a parameter, whose form is as follows (Zheng and Kong 2010):

3 $$h = \left\{ {\begin{array}{l} {\sigma_{ij} \frac{\partial g}{{\partial \sigma_{ij} }}} \hfill \\ {\delta_{ij} \frac{\partial g}{{\partial \sigma_{ij} }}} \hfill \\ {\left( {\frac{\partial g}{{\partial \sigma_{ij} }} \cdot \frac{\partial g}{{\partial \sigma_{ij} }}} \right)^{{\frac{1}{2}}} } \hfill \\ \end{array} } \right.\quad {\text{if}}\quad \begin{array}{l} {k = w^{p} } \\ {k = \theta^{p} } \\ {k = \bar{\varepsilon }^{p} } \\ \end{array}$$

where *w*^*p*^ is the plastic power, *θ*^*p*^ is the plastic expansion, $\bar{\varepsilon }^{p}$ is the equivalent plastic strain.

The switch function $\langle \cdot \rangle$ describes the loading and unloading criterion, whose form is as follows (Zheng and Kong 2010):

4 $$\left\langle {\frac{\partial F}{{\partial \varepsilon_{ij} }}d\varepsilon_{ij} } \right\rangle \left\{ {\begin{array}{l} > 0\quad {\text{loading}} \hfill \\ = 0\quad {\text{neural}}\,{\text{variable}}\,{\text{loading}} \hfill \\ < 0\quad {\text{unloading}} \hfill \\ \end{array} }\right.$$

The relationship of the two yield functions is as follows:

5 $$\frac{\partial F}{{\partial \varepsilon_{ij} }} = D_{ijkl} \frac{\partial f}{{\partial \sigma_{ij} }}$$

Equation (1) can be summarized by a matrix as

6 $$\{ d\sigma \} = \left\{ {\begin{array}{l} [D_{e} ]\{ d\varepsilon \} \hfill \\ ([D_{e} ] - [D_{p} ])\{ d\varepsilon \} \hfill \\ \end{array}} \right.\begin{array}{ll} \quad {if} \hfill & {f(\sigma ,\sigma^{p} ,k) < 0} \hfill \\ \quad{or} \hfill & {f(\sigma ,\sigma^{p} ,k) = 0} \hfill \\ \quad{if} \hfill & {f(\sigma ,\sigma^{p} ,k) = 0} \hfill \\ \end{array} \begin{array}{ll} {\text{and}} & {\left( {\frac{\partial f}{\partial \sigma }} \right)^{t} D_{e} d\varepsilon \le 0} \\ {\text{and}} & {\left( {\frac{\partial f}{\partial \sigma }} \right)^{t} D_{e} d\varepsilon > 0} \\ \end{array}$$

where *D*_*p*_ is a plastic matrix, *D*_*ep*_ is the elastic–plastic matrix [*D*_*ep*_] = [*D*_*e*_] − [*D*_*p*_].

Therefore, the elastic–plastic constitutive model law can be described by a plastic matrix. According to the mechanics derivation (Koichi 2009), the plastic matrix [*D*_*p*_] can be described as

7 $$[D_{p} ] = \frac{{\left\{ {\frac{\partial \varPhi }{\partial \sigma }} \right\}^{T} [D_{e} ][D_{e} ]^{T} \left\{ {\frac{\partial G}{\partial \sigma }} \right\}}}{{A + \left\{ {\frac{\partial \varPhi }{\partial \sigma }} \right\}^{T} [D_{e} ]\left\{ {\frac{\partial G}{\partial \sigma }} \right\}}}$$

where *Φ* is the loading function, *G* is the plastic potential.

In this study, the surrounding rock is supposed as perfect elastic–plastic. Therefore, its elastic–plastic constitutive model can be simply described as follows (Nakai 2012):

8 $$\{ d\sigma \} = \left\{ {[D_{e} ] - \frac{{[D_{e} ]\left\{ {\frac{\partial G}{\partial \sigma }} \right\}\left\{ {\frac{\partial F}{\partial \sigma }} \right\}^{T} [D_{e} ]^{T} }}{{\left\{ {\frac{\partial F}{\partial \sigma }} \right\}^{T} [D_{e} ]\left\{ {\frac{\partial G}{\partial \sigma }} \right\}}}} \right\}\{ d\varepsilon \}$$

*F* can be described as (Zheng et al. 2002)

9 $$F(I_{1} ,J_{2} ,J_{3} ) = 0$$

where *I*_1_ is the first invariant of stress, *J*_2_ is the second invariant, *J*_3_ is the third invariant of the stress deviator.

For the non-associative flow rule, the plastic potential *G* is different from the yield function *F*. And it is a very hard work to construct the function of *G* (Nakai 2012). Moreover, there is no generalized form for the function of *G* (Zheng et al. 2002). However, the elastic–plastic constitutive model with the associative flow rule can describe the main mechanical behaviours of geo-meterials well (Zheng and Kong 2010). Thus, in this study, simply, *G* = *F*.

Therefore, only two terms are to be determined in this elastic–plastic constitutive law: the stress yield function *F* and the elastic matrix [*D*_*e*_]. The problem of identification for the elastic–plastic constitutive model can be transformed to the problem of identification for the stress yield function *F* and the elastic parameters *E* and *μ*.

The generalized form of the stress yield function is (Zheng et al. 2002)

10 $$F = \beta I_{1}^{2} + \alpha I_{1} - K + \bar{\sigma }_{ + }^{n} = 0$$

where $\bar{\sigma }_{ + }^{n} = \frac{{\sqrt {J_{2} } }}{s(\theta )}$, *s*(*θ*) is a function to describe the shape of yield function (Liu and Zheng 1997; Zheng and Kong 2010), *θ* is the Lode angle, *α*, *β* and *K* are unknown parameters.

**Fig. 3:**
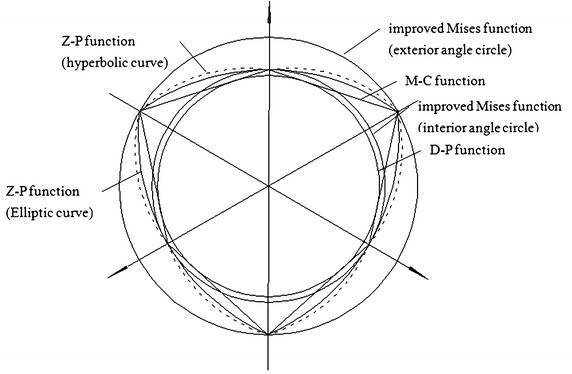
Some yield function curves by the generalized form of the yield function

 Because the Eq. (10) is the generalized form of the yield functions, with the different parameters, mainly *α* and *K*, the different yield functions can be obtained (Zheng et al. 2002). These yield functions include almost all widely used yield functions, such as Mohr–Coulomb (M–C) yield criterion, Mises yield criterion, two improved Mises yield criterions (exterior angle circle and interior angle circle), Drucker–Prager (D–P) yield criterion, Tresca yield criterion, three Zienkiewicz–Pande (Z–P) yield criterions (elliptic curve, hyperbolic curve and parabolic curve) and twin-shear yield criterion, etc. The detailed specific relationship between different parameters and different yield functions can be found in reference (Zheng et al. 2002). The curves of the Eq. (10) for some generally used yield functions are shown in Fig. 3.

Using this generalized constitutive law for an elastic–plastic model, the problem of model identification can be transformed to a parameter identification.

To simply analyze without a loss of generalization, we assume that the parameter *β* = 0; thus, only the two parameters *α* and *K* need to be identified. In addition to the elastic parameters *E* and *μ*, four model parameters need to be identified: *E*, *μ*, *α* and *K*.

## Model identification by immunized genetic algorithm

**Fig. 4:**
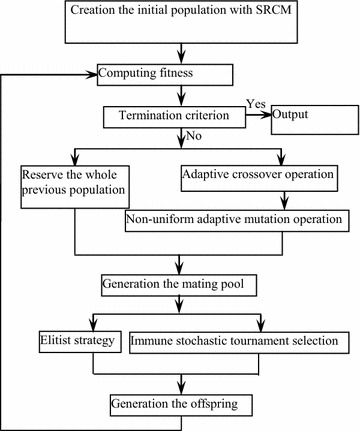
Flow chart of immunized genetic algorithm

To improve the main operations of genetic algorithm, such as creation of initial population, reproduction operation, crossover operation, mutation operation and selection operation, one new immunized genetic algorithm has been proposed by author, whose flow chart is as shown in Fig. 4. The details of the immunized genetic algorithm can be found in reference (Gao 2012).

**Fig. 5:**
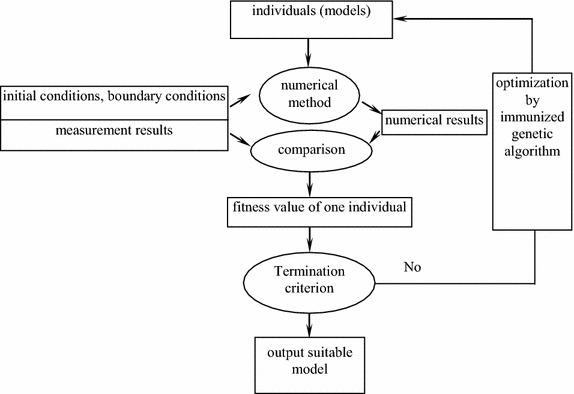
Flow chart of model identification based on immunized genetic algorithm

Based on new immunized genetic algorithm, the model identification method can be proposed, whose flow chart is as shown in Fig. 5.

The detailed procedure of the model identification is described in the following.

1. Parameter initialization

Before the algorithm is performed, all parameters must be initialized. These parameters include the number of individuals in the initial population, the maximum number of iterations and the ranges for the inversed model parameters, which are Young’s Modulus *E*, Poisson’s ratio *μ*, model parameters *α* and *K*.

2. Individual expression

In this study, an individual is expressed as *X* = (*E*, *μ*, *α*, *K*). An individual corresponds to an elastic–plastic constitutive model.

3. Creation of the initial population

Initial population is created by SRCM (Gao and Yin 2011).

4. Fitness function

In this study, the objective function is defined as

11 $$f(i) = \sum\limits_{i = 1}^{Num} {\left| {e(i)} \right|}$$

where *e*(*i*) is the error between the displacement computations and the displacement measurements and *Num* is the number of monitor points.

The fitness function is as follows:

12 $$F(i) = 1.0/(1.0 + f(i))$$

5. Optimization process

The optimization operation is as same as in immunized genetic algorithm (Gao 2012).

6. Termination condition

In this study, the termination criterion specifies that the difference of the maximum fitness value and the average fitness value is less than 10e-5. To avoid infinite iteration, a maximum number of evolutionary generations is also specified.

## Case study

### Numerical example

A numerical testing example is employed to test the proposed algorithm. The example is an underground tunnel with a radius of 3 m. The details of this example can be found in reference (Gao 2016).

For computation, the mechanical property of the surrounding rock is assumed as follows:

The surrounding rock is an elastic–plastic material. The associated parameters are Young’s Modulus *E* = 2100 MPa, Poisson’s ratio *μ* = 0.2, cohesion *C* = 1.1 MPa and friction angle $\varphi$ = 30°. Because the yield function is the Drucker–Prager theory, the definitions of *α* and *K* (Zheng et al. 2002) are as follows,

13 $$\alpha = \frac{3\sin \varphi }{{\sqrt 3 \sqrt {3 + \sin^{2} \varphi } }}$$

14 $$K = \frac{3C\sin \varphi }{{\sqrt 3 \sqrt {3 + \sin^{2} \varphi } }}$$

According to the definition of *α* and *K*, we can obtain the values of *α* and *K* from the values of *C* and $\varphi$, which are as follows: *α* = 0.165872, *K* = 0.875726 MPa.

Because the ranges of the parameters only affect the computing efficiency and have minimal influence on the computing results, the ranges for the four parameters are as follows:

$$2000\,{\text{MPa}} \le E \le 2400\,{\text{MPa}},\quad 0.1 \le \mu \le 0.3,\quad 0.0 \le \alpha \le 0.6,\quad 0.0 < K \le 2.5\,{\text{MPa}}.$$

For comparison study, the traditional genetic algorithm (GA), fast genetic algorithm (FGA) (Gao 2007) and the immunized genetic algorithm (IGA) are all applied for this example.

Based on testing and experience, the parameters for three algorithms are summarized in Table 1.

**Table 1:** Parameters for three algorithms (GA, FGA and IGA)

	*n*	*m*	*p*_c_	*p*_m_
GA	100	500	0.65	0.15
FGA	100	500	–	–
IGA	100	500	–	–

*n* is the number of individuals, *m* is maximum number of evolutionary generations, *p*_c_ is the probability of crossover and *p*_m_ is the probability of mutation

To compare the computation effect, the results of GA, FGA and IGA are summarized in Table 2.

**Table 2:** Comparison of model identification results

	*E*/MPa	*μ*	*α*	*K*/MPa
Theory values	2157	0.2	0.165872	0.875726
Identified values by GA	2135	0.182	0.171365	0.853471
Relative error of computing results for GA (%)	1.02	9	3.31	2.54
Identified values by FGA	2140	0.182	0.168442	0.853532
Relative error of computing results for FGA (%)	0.79	9	1.55	2.54
Identified values by IGA	2143	0.188	0.167812	0.854621
Relative error of computing results for IGA (%)	0.65	6	1.17	2.41

As shown in Table 2, the results of the IGA are superior to the results of the GA and FGA; therefore, the computation effect in this study is reasonable.

**Fig. 6:**
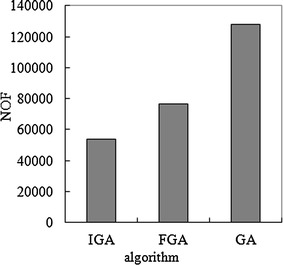
Comparison of NOFs for GA, FGA and IGA

To simply evaluate the computational efficiency of the GA, FGA and IGA, the number of objective function evaluations during the search for the optimum, which is denoted by NOF and represents the computation time required by the optimization algorithm, is applied. The NOFs of the GA, FGA and IGA methods are given in Fig. 6.

As shown in Fig. 6, the NOF of IGA is much less than those of other algorithms, and then the computational speed of IGA in this study is very faster than those of other algorithms in the literature. In other words, the computational efficiency of the IGA in this study is the best and is superior to those of other algorithms.

The results of these studies conclude that the IGA method can be used to obtain a suitable model with higher accuracy and less effort.

### Engineering example

The Huainan coal mine is located north of Huainan city, of Anhui Province in China. As an old mining, the entire mine has been integrated into the stage of deep mining. The different geological environments range from shallow rock roadway to the deep rock roadway; as a result, the mechanical characteristics are very complicated. To analyze the failure mechanism of the surrounding rock for deep rock roadway, a constitutive model of the surrounding rock based on the displacement measurement results must be identified. In this study, two main types of surrounding rock are identified: types III and II (Liu et al. 2010).

**Fig. 7:**
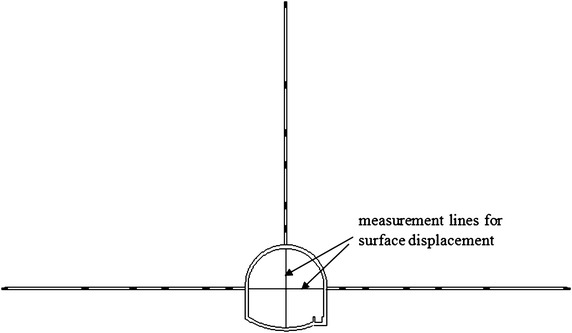
Layout of the monitoring points for −780 $C_{13}^{S}$ floor haulage roadway in the Xieyi mine

The surrounding rock of the −780 $C_{13}^{S}$ floor haulage roadway in the Xieyi mine is characterized as the moderate poor type, which is associated with type III. The depth of this roadway is 800 m. Its main lithology consists of fine sandstone and limestone, and its integrity is satisfactory. The roadway cross-section is shown in Fig. 7. Its width is 3.8 m, the height of the side wall is 1.3 m, the height of the crown is 1.9 m and the depth of the floor arch is 0.5 m.

To analyze the stability of the roadway, some site monitoring studies have been performed, including surface displacement measurements and deep displacement measurements.

For the surface displacement measurements, the convergence between both sides and the convergence between the roof and the floor were completely measured. The layout of the monitoring points is illustrated in Fig. 7. The layout of the monitoring points for the deep displacement measurements is also illustrated in Fig. 7. The depth of each monitoring hole was 15 m. Six monitoring points were installed along each monitoring hole, with distances of 1, 3, 5, 7, 10 and 15 m, which are designated point 1, point 2, point 3, point 4, point 5 and point 6. Assuming the point at a depth of 15 m is stationary, the relative displacement of the remaining points can be obtained.

**Fig. 8:**
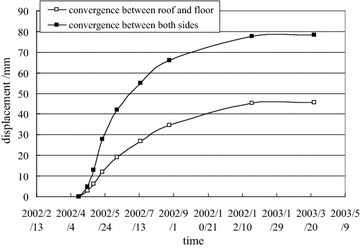
Measurement of surface convergence displacement of −780 $C_{13}^{S}$ floor haulage roadway in the Xieyi mine

**Fig. 9:**
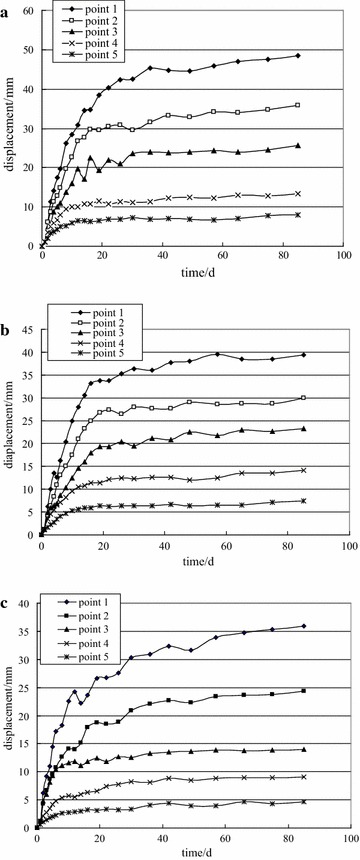
Measurement of deep multi-point displacement of −780 $C_{13}^{S}$ floor haulage roadway in the Xieyi mine. **a** Top, **b** left side, **c** right side

The surface convergence displacement measurement and deep multi-point displacement are shown in Figs. 8 and 9.

**Fig. 10:**
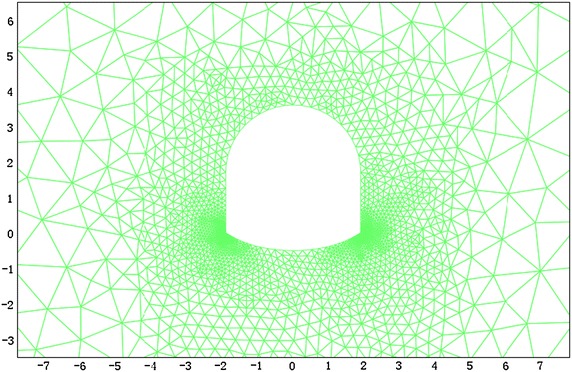
FEM computation model for −780 $C_{13}^{S}$ floor haulage roadway in the Xieyi mine

According to the field monitoring of the hydraulic fracturing technique, the vertical initial stress is 13.2 MPa and the horizontal initial stress is 19.5 MPa. The FEM model is shown in Fig. 10.

Because the ranges of the parameters only affect the computing efficiency and have minimal influence on the computing results, the ranges of the four model parameters are as follows:

$$1\,{\text{GPa}} \le E \le 25\,{\text{GPa}},\quad 0.15 \le \mu \le 0.35,\quad 0.0 \le \alpha \le 0.8,0.0 < K \le 3.5\,{\text{MPa}}.$$

Based on the surface convergence displacement, the constitutive model of the surrounding rock can be performed by this new method.

The parameters of model identification method are as follows:

The number of individuals is 150, and the maximum number of evolutionary generations is 500.

The identified model parameters are as follows:

$$E = 12.6\,{\text{GPa}},\quad \mu = 0.3,\quad \alpha = 0.2056, \quad K = 0.9216\,{\text{MPa}}.$$

In previous studies (Liu et al. 2010), based on some laboratory experiments and engineering experience, the suggestion values for mechanical parameters of typical surrounding rock were given to conduct the support design. Because the suggestion values can consider the complicated geological environment for surrounding rock, they are very suitable to be used to test the identified parameters in this study. The suggestion values of mechanical parameters for surrounding rock of type III are as follows (Liu et al. 2010).

*E* is between 6 and 15 GPa, *μ* is almost 0.3, *C* is between 1.0 and 1.5 MPa and $\varphi$ is between 30° and 45°.

Thus, the identified values for parameters *E* and *μ* are agree with the suggestion values well. However, the two model parameters *α* and *K* can not be obtained directly from the tests, and they can be determined from the parameters *c* and *φ* based on the yield function. To obtain the model parameters, the yield function of the surrounding rock must be constructed, but it is a very hard work. Because this study is only to analyze the stability of surrounding rock, the hard work to construct the yield function of the surrounding rock is not conducted. Therefore, the model parameters *α* and *K* can only be verified by the comparison of measured deep multi-point displacements and the computed deep multi-point displacements by FEM with the identified model.

**Fig. 11:**
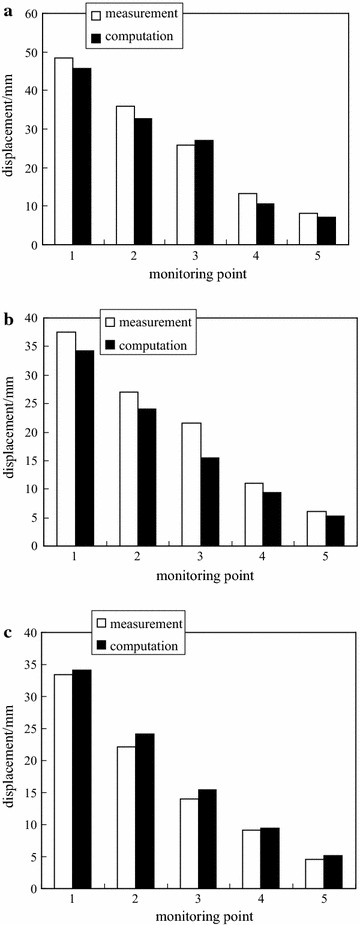
Comparison of deep multi-point displacements of −780 $C_{13}^{S}$ floor haulage roadway in the Xieyi mine. **a** Top, **b** left side, **c** right side

To verify the model identification results, the deep multi-point displacements can be computed by the FEM with the identified model. The results are shown in Fig. 11. For comparison, the stable displacement of each monitoring point is utilized.

As shown in Fig. 11, the computation displacements correspond with the measured displacements. Therefore, the identified model for the surrounding rock of the −780 $C_{13}^{S}$ floor haulage roadway in the Xieyi mine can adequately describe the real rock performance.

**Fig. 12:**
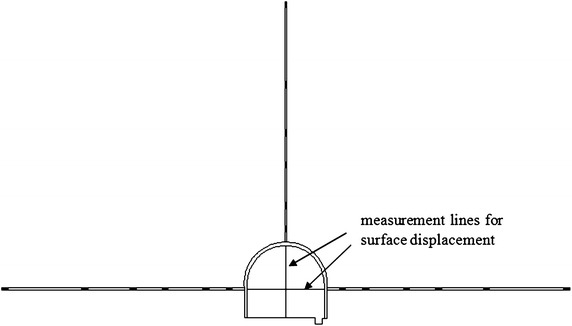
Layout of the monitoring points for −720 rock level crosscut in the Xieqiao mine

The surrounding rock of the −720 rock level crosscut in the Xieqiao mine is the moderate type, which corresponds to type II. The depth of this roadway is 720 m. Its main lithology consists of fine sandstone, fine siltstone and medium fine sandstone, and its integrity is satisfactory. The cross-section of the roadway shown in Fig. 12. Its width is 4.5 m, the height of the side wall is 1.5 m and the height of the crown is 2.25 m.

The layout of the monitoring points is illustrated in Fig. 12. The layout of the monitoring points for the deep displacement measurements is also illustrated in Fig. 12. The depth of each monitoring hole was 15 m. Six monitoring points were installed along each monitoring hole, with distances of 1, 3, 5, 7, 10 and 15 m, which are designated point 1, point 2, point 3, point 4, point 5 and point 6. Assuming that the point at a depth of 15 m is stationary, the relative displacement of the remaining points can be obtained.

**Fig. 13:**
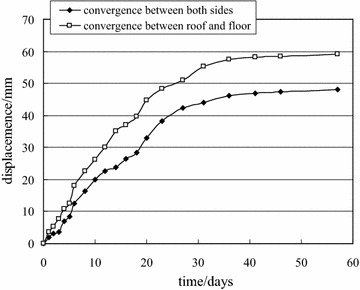
Measurement of surface convergence displacement of −720 rock level crosscut in the Xieqiao mine

**Fig. 14:**
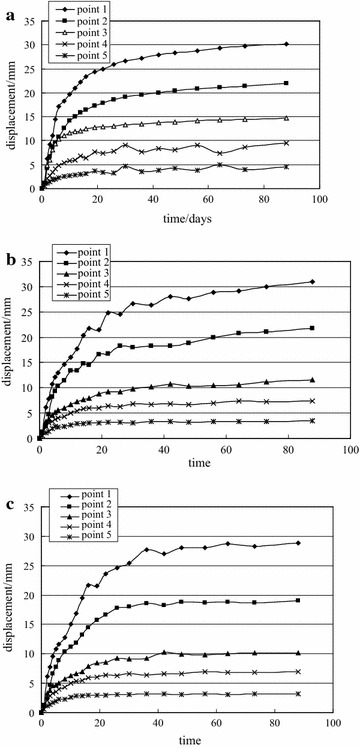
Measurement of deep multi-point displacement of −720 rock level crosscut in the Xieqiao mine. **a** Top, **b** left side, **c** right side

The surface convergence displacement measurement and deep multi-point displacements are shown in Figs. 13 and 14.

**Fig. 15:**
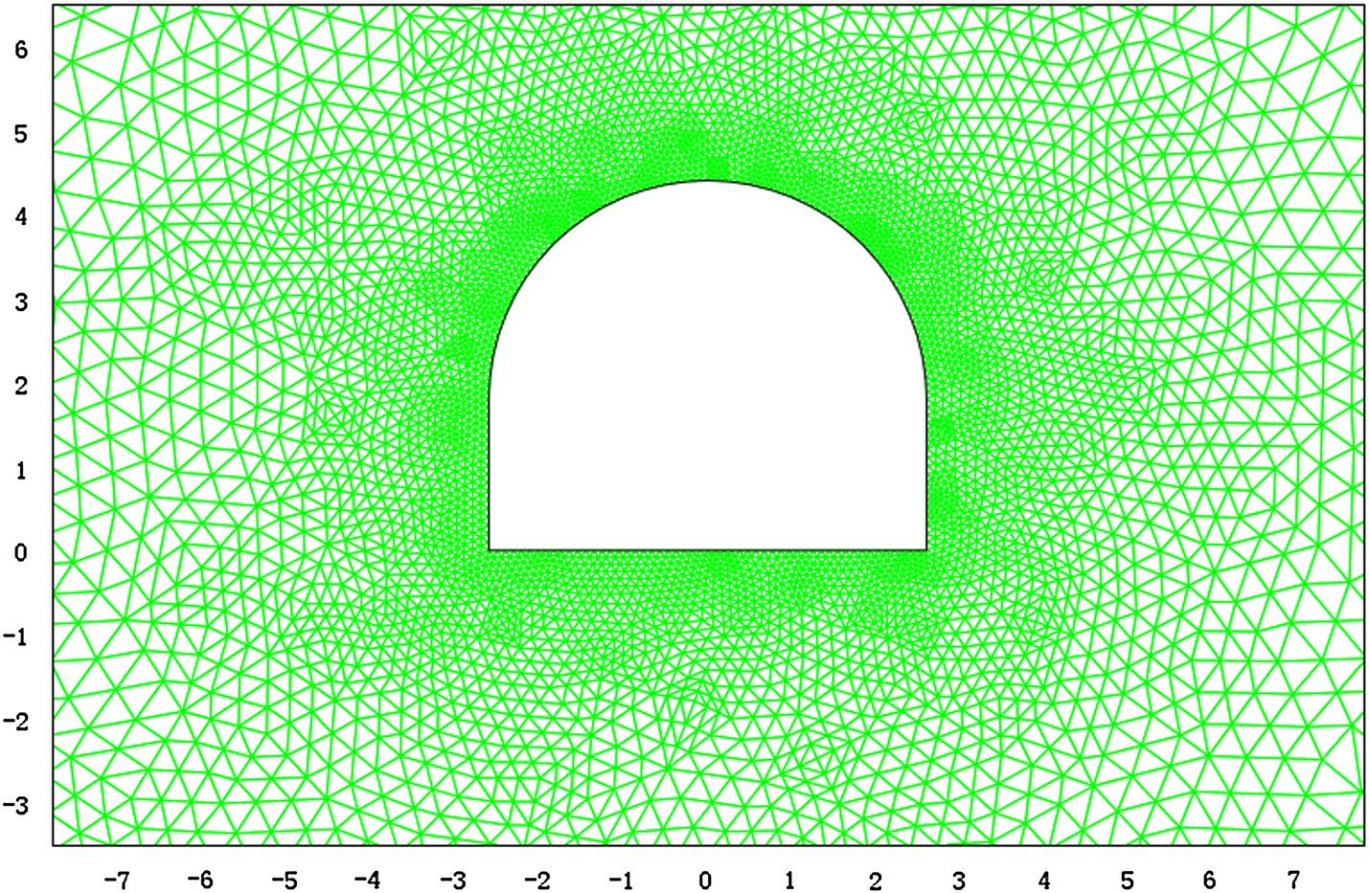
FEM computation model for −720 rock level crosscut in the Xieqiao mine

According to the field monitoring results, the vertical initial stress is 20.1 MPa and the horizontal initial stress is 15.6 MPa. The FEM model is shown in Fig. 15.

Because the ranges of the parameters only affect the computing efficiency and have minimal influence on the computing results, the ranges for the four model parameters are as follows:

$$1\,{\text{GPa}} \le E \le 30\,{\text{GPa}},\quad 0.15 \le \mu \le 0.35,\quad 0.0 \le \alpha \le 0.85,\quad 0.0 < K \le 4.5\,{\text{MPa}}.$$

Based on the surface convergence displacement, the constitutive model of the surrounding rock can be performed by this new method.

The parameters of model identification method are as follows:

The number of individuals is 150, and the maximum number of evolutionary generations is 500.

The identified model parameters are as follows:

$$E = 14.6\,{\text{GPa}},\quad \mu = 0.26,\quad \alpha = 0.1871,\quad K = 0.8841\,{\text{MPa}}.$$

As the same studies for surrounding rock of type III, the two mechanical parameters *E* and *μ* are verified by the suggestion values of mechanical parameters in previous study and the model parameters *α* and *K* are verified by measured deep multi-point displacements.

The suggestion values of mechanical parameters for surrounding rock of type II are as follows (Liu et al. 2010).

*E* is between 10 and 25 GPa, *μ* is almost 0.25, *C* is between 1.2 and 2.0 MPa and *φ* is between 40° and 55°.

Thus, the identified values for parameters *E* and *μ* are agree with the suggestion values well. The model parameters *α* and *K* are verified by the comparison of measured deep multi-point displacements and the computed deep multi-point displacements by FEM with the identified model.

**Fig. 16:**
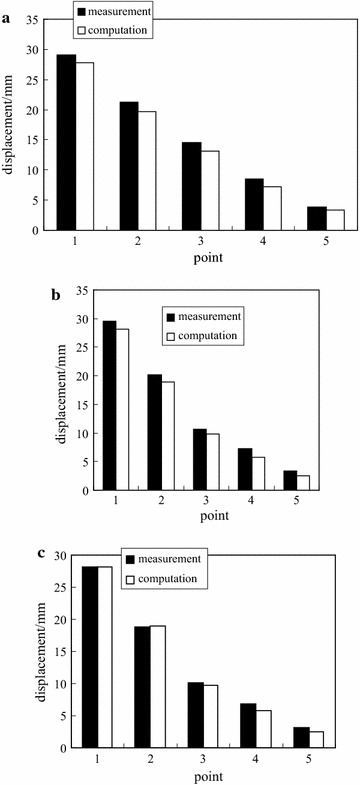
Comparison of deep multi-point displacements of −720 rock level crosscut in the Xieqiao mine. **a** Top, **b** left side, **c** right side

To verify the model identification results, the deep multi-point displacements can be computed by the FEM with the identified model. The results are shown in Fig. 16. For comparison, the stable displacement of each monitoring point is employed.

As shown in Fig. 16, the computation displacements coincide with the measured displacements. Therefore, the identified model for the surrounding rock can adequately describe the real rock performance.

In this study, the surrounding rock is assumed as one equivalent homogeneous material. Therefore, the computed displacements at both sides are equal. In fact, the measurement displacements at both sides are different. In other words, the inhomogeneous displacement as shown in Figs. 11 and 16 can not be described in this study.

The engineering applications prove that the new model identification method proposed in this study can be used to identify a suitable constitutive model for the surrounding rock of the underground engineering with reasonable efficiency using only surface displacement measurements.

## Conclusion

The identification of a rock constitutive model is very important. From the theory analysis of the elastic–plastic constitutive law of rock materials, the generalized law of constitutive model is presented. Using this generalized law, the problem of identification of a constitutive model can be transformed to parameter identification. Therefore, the new immunized genetic algorithm proposed by the author is applied to an elastic–plastic model identification problem. Using a numerical example and an engineering example, this new method is verified. The results indicate that the proposed method can be used to identify a suitable constitutive model with high accuracy and efficiency.
